# In‐vivo quality assurance of dynamic tumor tracking (DTT) for liver SABR using EPID images

**DOI:** 10.1002/acm2.13969

**Published:** 2023-03-30

**Authors:** Maryam Rostamzadeh, Kurt Luchka, Roy Ma, Mitchell Liu, Emma Dunne, Marie‐Laure Camborde, Tania Karan, Ante Mestrovic, Alanah Bergman

**Affiliations:** ^1^ Department of Physics and Astronomy University of British Columbia Vancouver BC Canada; ^2^ Medical Physics Department BC Cancer‐Vancouver Vancouver Canada; ^3^ Radiation Oncology Department BC Cancer‐Vancouver Vancouver Canada

**Keywords:** detection algorithm, dynamic tumor tracking, EPID, marker detection, position verification

## Abstract

**Purpose:**

To assess dynamic tumor tracking (DTT) target localization uncertainty for in‐vivo marker‐based stereotactic ablative radiotherapy (SABR) treatments of the liver using electronic‐portal‐imaging‐device (EPID) images. The Planning Target Volume (PTV) margin contribution for DTT is estimated.

**Methods:**

Phantom and patient EPID images were acquired during non‐coplanar 3DCRT‐DTT delivered on a Vero4DRT linac. A chain‐code algorithm was applied to detect Multileaf Collimator (MLC)‐defined radiation field edges. Gold‐seed markers were detected using a connected neighbor algorithm. For each EPID image, the absolute differences between the measured center‐of‐mass (COM) of the markers relative to the aperture‐center (Tracking Error, (E_T_)) was reported in pan, tilt, and 2D‐vector directions at the isocenter‐plane.

**Phantom study:**

An acrylic cube phantom implanted with gold‐seed markers was irradiated with non‐coplanar 3DCRT‐DTT beams and EPID images collected. *Patient Study*: Eight liver SABR patients were treated with non‐coplanar 3DCRT‐DTT beams. All patients had three to four implanted gold‐markers. In‐vivo EPID images were analyzed.

**Results:**

*Phantom Study*: On the 125 EPID images collected, 100% of the markers were identified. The average ± SD of E_T_ were 0.24 ± 0.21, 0.47 ± 0.38, and 0.58 ± 0.37 mm in pan, tilt and 2D directions, respectively. *Patient Study*: Of the 1430 EPID patient images acquired, 78% had detectable markers. Over all patients, the average ± SD of E_T_ was 0.33 ± 0.41 mm in pan, 0.63 ± 0.75 mm in tilt and 0.77 ± 0.80 mm in 2D directions The random 2D‐error, σ, for all patients was 0.79 mm and the systematic 2D‐error, Σ, was 0.20 mm. Using the Van Herk margin formula 1.1 mm planning target margin can represent the marker based DTT uncertainty.

**Conclusions:**

Marker‐based DTT uncertainty can be evaluated in‐vivo on a field‐by‐field basis using EPID images. This information can contribute to PTV margin calculations for DTT.

## 1 INTRODUCTION

In radiation therapy, intrafractional motion is one of the most intractable sources of uncertainty for tumors located in the thoracic and abdominal regions.^1^, ^2^ Various motion management methods have been proposed for treating moving tumors to reduce the effect of intrafractional motion on the radiation dose delivery. The most recent approach, Real‐time Dynamic Tumor Tracking (DTT), has the radiation beam following the tumor motion throughout the entire respiratory cycle.^1^, ^2^, ^3^ Several DTT technologies have been developed; such as robotic‐arm controlled linacs,^4^ gimbal‐mounted linacs,^5^, ^6^ conventional linacs employing dynamic multi‐leaf collimators (MLC),^7^, ^8^, ^9^ helical‐motion linacs combining MLC + jaw motions,^10^ and conventional linacs with dynamic couches that move the patient.^11^, ^12^

To perform DTT safely and accurately, quantifying the residual uncertainty of the tracking system is important.

Several authors have suggested using electronic portal imaging devices (EPIDs) to quantify marker‐based DTT uncertainty.^5^, ^13^, ^14^ The concept of detecting implanted markers on EPID images has been around for many years, and authors have offered various processing techniques to detect and quantify marker locations on the lower‐contrast portal images.

Early on in the introduction of MV portal imaging, Nederveen et al.^15^ developed a fiducial marker detection method based on a Marker Extraction Kernel (MEK). They studied the detection success rate for gold markers placed on the skin surface of patients undergoing lateral 18 MV beams as part of their prostate cancer treatment. Portal images using a camera‐based portal imaging device were collected. The detection success rate for markers (5 mm length, 1.2 mm diameter) was 39% when marker was placed at the beam exit side of the patient.

EPIDs have since been used to monitor markers during radiotherapy and to verify treatment target positions in both static and clinical respiratory correlated DTT deliveries.

Park et al.^16^ reported on an in‐vivo, EPID based marker detection method and calculated the 3D marker position on cine MV images of patient liver 3DCRT stereotactic ablative radiotherapy (SABR) treatments. They used a Laplacian of Gaussian (LoG) filter and rigid distances between the markers calculated from the planning CT to support the localization algorithm. In phantom, the detection success rate was as high as 88.8% which increased to 100% when they used prior CT information. In the patient study, the marker detection success rate was limited by organ deformation, couch rotations, MLC blocking, or when there were two markers were close to each other.

Azcona et al.^17^ also implemented a LoG filter with a normalized cross correlation method and template matching using a priori knowledge of MV beam apertures and marker (5 mm length, 1 mm diameter) locations projected from the treatment planning system. MV images collected during five prostate VMAT treatments demonstrated a marker detection success rate of 23%–65% (for all markers), and up to 40%–95% (for at least one marker). Modulating MLC leaves contributed to the limitation in the marker detection success rate.

In the context of dynamic tumor tracking (DTT), Keall et al.^13^ developed and evaluated a near real‐time EPID based marker (3 mm length, 1 mm diameter) tracking system intended for eventual real time DTT verification applications. The method involved manually defining a region‐of‐interest on an initial EPID image and applying a morphological opening operation sensitive to specific structure element shapes. Further denoising (Weiner) and smoothing (median) filters were applied to highlight the marker positions. In‐phantom results indicated that marker segmentation could be applied in 10−30 ms of computing time. Cho et al.^14^ report on a marker (3 mm length, 1 mm diameter) segmentation and localization method using simultaneous kV‐MV imaging systems for dynamic MLC tumor tracking applications. A limited field‐of‐view template matching algorithm is used to detect the marker in the EPID image, and the information is used as both input into the MLC tracking algorithm and as a method to quantify the tracking uncertainty. The average tracking error reported in phantom for an elliptical motion (2 cm superior‐inferior/1 cm left‐right, 10 cycles/min) pattern was 0.9 ± 0.5 mm. The main time limitation for real‐time applications was in the image acquisition, handling, and processing time (400 ms).

Poels et al.^18^ reported tracking errors based on two mutually independent systems available on‐board on the Vero4DRT system: log files combined with stereoscopic kV X‐ray imaging and EPID fluoroscopy. EPID images were processed with pulsed MV beam artifact suppression and LoG filtering. Normalized cross correlation (NCC) was used with limited‐region‐of‐interest template matching to locate the markers. Similarly, the tracking field center was located with a NCC method using a reference field shape obtained from the planning system. For the one liver cancer patient, gimbaled tracking showed a mean/90th percentile absolute tracking error of 0.76 mm/2.81 mm and 0.32 mm/2.24 mm in the tilt and pan direction based on EPID fluoroscopy images.

Finally, Akimoto et al.^5^ assessed the long‐term stability of DTT errors on the Vero4DRT system (BrainLAB AG, Feldkerchen, Germany/Mitsubushi Heavy Industries, Tokyo, Japan) using a 1D sinusoidal respiratory motion platform with a square phantom embedded a steel ball at center. EPID images were collected and analyzed. This group report an average value of maximum deviation between the center of the image of the steel ball and the center of the MV field for a single moving anterior beam (±10 mm motion, 10 cycles/min) of 0.38, 0.49, and 0.53 mm in pan, tilt and 2D directions, respectively.

This study presents in‐vivo, EPID measured marker‐based DTT uncertainties using the Vero4DRT radiotherapy system for thirteen liver SABR deliveries. This retrospectively collected information can be used to calculate and validate appropriate PTV margins for this delivery technique.

## 2 METHODS AND MATERIALS

### 2.1 Vero4DRT radiotherapy linear accelerator

**FIGURE 1:**
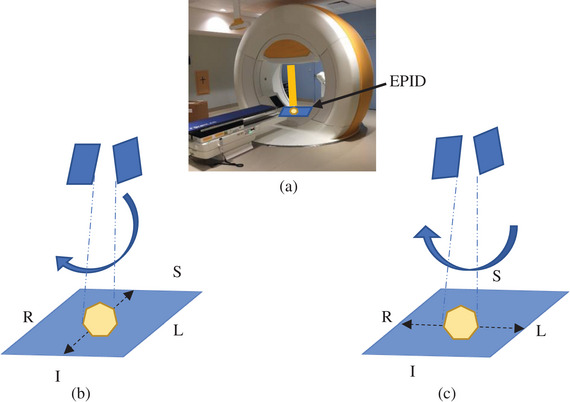
(a) Vero4DRT gimballed linear accelerator EPID location (blue plane) for Gantry = 0^o^, Ring = 0^o^. Treatment field motions in (b) tilt and (c) pan directions. The EPID is 221.2 cm from the X‐ray target. Tracking motion is reported in millimeters along the EPID plane, de‐magnified to isocenter (100 cm from target). S/I = superior/inferior & R/L = Right/Left (note: patient co‐ordinates in this figure applies to Gantry = 0^o^, Ring = 0^o^ only).

The Vero4DRT linear accelerator is a dedicated SABR accelerator, built into a “O”‐ring geometry and capable of DTT.^6^, ^19^, ^20^ Tracking is achieved by mounting the waveguide/treatment head onto a 2D gimbal capable of pan/tilt motions (Figure 1). This linear accelerator generates a 6 MV flattened photon beam (500 MU/min maximum dose rate at 100 cm SAD). During the marker based DTT workflow, the motion of an external surrogate (chest IR marker pad) is correlated with the center‐of‐mass of the internal target (or marker surrogate) motion. The internal motion is detected via an integrated, dual‐orthogonal X‐ray fluoroscopy system (ExacTrac, BrainLAB AG, Feldkerchen, Germany). The fiducial location and geometric pattern/distribution are auto detected on the kV images using a priori knowledge originating from the CT planning scan which has been imported into ExacTrac. Once a correlation model is established, the DTT motion is determined by monitoring the external surrogate. The Vero4DRT aligns the beam aperture to the detected fiducial center‐of‐mass (COM). The detected versus predicted fiducial locations and their COM is verified throughout the tracked beam delivery. The beam will automatically be withheld if deviations beyond a user‐defined limit are exceeded (typically set to 3 mm for clinical applications).

The Vero4DRT is equipped with an integrated, amorphous silicon EPID. Source Detector Distance (SDD) is 221.2 cm. The EPID system has a matrix size of 1024 × 1024 pixels and pixel size of 0.18 × 0.18 mm^2^ (de‐magnified to the isocenter plane).^5^ The EPID system has a maximum frame rate of 2 Hz. Vero4DRT passively acquires EPID images during every radiation beam delivery and stores the images in a 1.2 MB “. raw” format. The pan and tilt tracking direction of the moving MV beam aperture is consistent relative to the EPID imager plane (see Figure 1).

### 2.2 Phantom study

**FIGURE 2:**
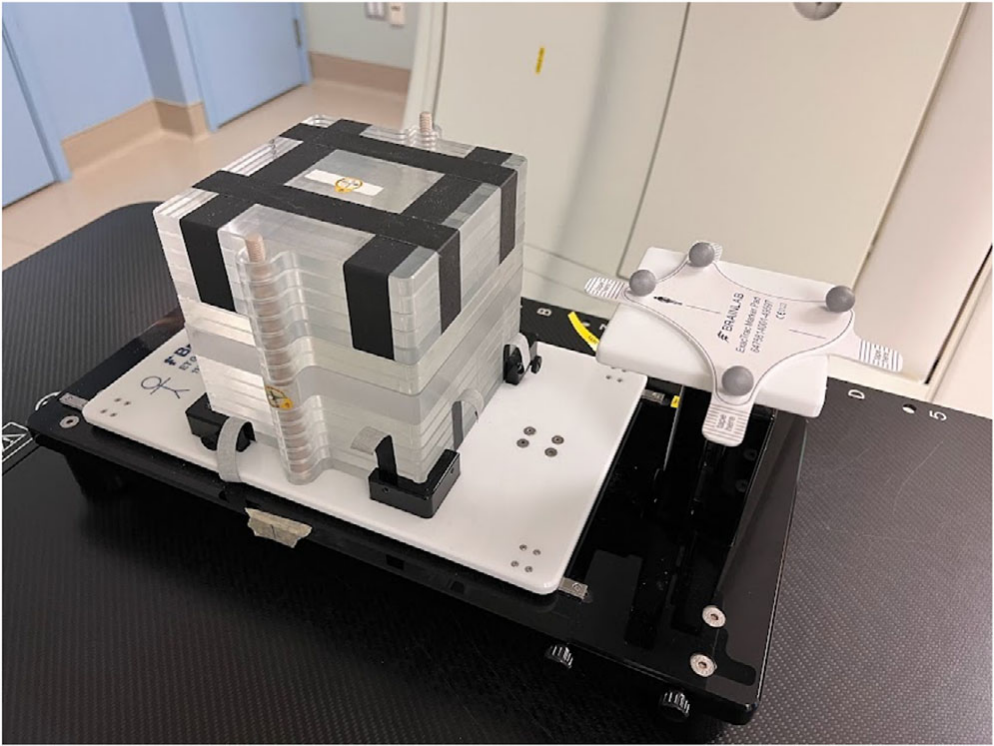
An acrylic multi slab phantom assembled to create a cubic geometry. Phantom is placed on a moving platform designed to simulate 1D phantom motion and orthogonal respiratory motion.

An acrylic, multi‐slab phantom (BrainLAB AG, Feldkerchen, Germany) was assembled to create a cubic geometry (Figure 2). Three gold markers (3 mm length, 1 mm diameter) are embedded into one of the slabs. The phantom was placed on a 1D programmable, moving platform (BrainLAB AG) comprised of a respiratory chest platform moving orthogonal (anterior/posterior) to the phantom motion (superior/inferior). A sinusoidal breathing trace (6 BPM, 20 mm amplitude) was used. Six non‐coplanar 3DCRT fields were delivered to the phantom while performing a marker based DTT workflow. EPID images (125 in total, approximately 21 images per beam) were acquired during beam delivery.

### 2.3 Patient study

This retrospective study included sequential SABR DTT patients treated between Aug 2018 and July 2020 by 3DCRT method. Three to four gold markers (3 mm length, 1 mm diameter) were implanted close to the liver tumor per standard procedures. Patient contouring and non‐coplanar 3D‐CRT planning was performed on a breath hold exhale CT simulation scan. During treatment, forty‐four 3DCRT plans were delivered. Of these, only 13 plans (eight patients) had EPID images collected and saved during treatment. The eligibility requirement for this study was: (1) patient must have been treated with 3DCRT (not IMRT), and (2) patient must have EPID images archived as part of their treatment. On average, 110 EPID images were obtained per treatment fraction (range: 54–290). Table 1 reports the patient characteristics and their SABR beam parameters.

**TABLE 1:** Liver SABR plan characteristics. Of the 1430 EPID patient images acquired, 78% had detectable markers

Patient ID	GTV volume (cc)	Total dose (Gy)	# Fractions	# Fields	# Fractions used for study	# EPIDs collected	# EPIDs analyzed
1	28.9	45	3	7	2	101	97
						100	94
2	16.4	45	3	8	3	90	73
						88	72
						88	83
3	11.0	54	3	7	1	104	104
4	2.3	45	3	7	1	290	229
5	70.0	45	3	7	1	139	89
6	45.0	45	3	11	3	54	54
						98	58
						104	78
7	6.4	45	3	9	1	78	28
8	27.5	45	5	9	1	96	66

### 2.4 Data analysis

**FIGURE 3:**
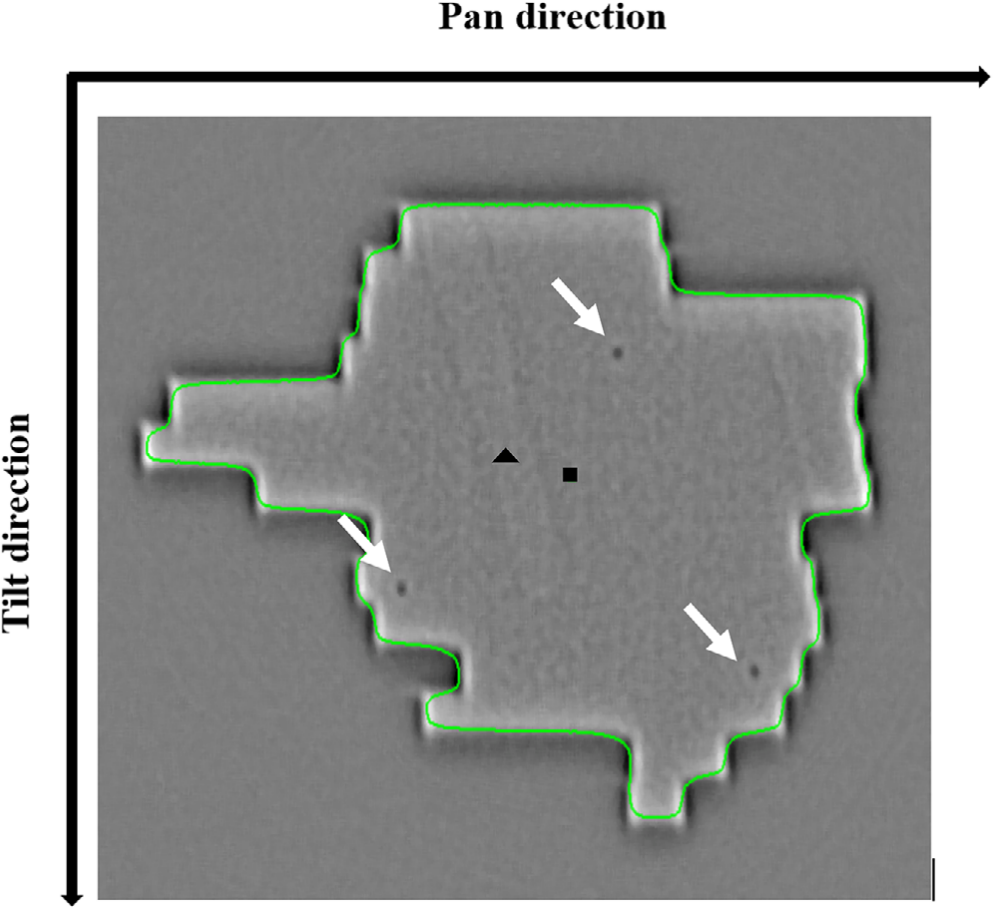
Processed EPID image from Vero4DRT Linac. Green line: detected field edge. Arrows: fiducial markers (×3). Triangle: Center of EPID imager. Square: Center‐of‐Mass of radiation field aperture.

Each EPID image (1024 × 1024 matrix) is converted to 256‐bit grayscale images with image data values ranging from 0 (white) to 1 (black). Images were preprocessed using a Laplacian of Gaussian (LoG) filter. In this study, a chain code algorithm^21^ has been applied to EPID images to detect the MLC defined radiation field edges (Figure 3). The geometric center of the aperture was calculated. The algorithm was implemented in JAVA (Oracle Corporation, Redwood, CA, USA). A connected neighbor marker detection algorithm^21^ was applied for identifying the 2D locations of the implanted markers in EPID images. The EPID is 221.2 cm from the X‐ray target. The difference between the center of the aperture and the COM of the markers in the pan/tilt directions on the EPID was first identified in pixel values. The pixel values were converted to absolute distance in mm at isocenter (100 cm) using the factor of 1 pixel = 0.18 mm. The tracking error (E_T_) is defined as the absolute variation in the relative distance between the aperture center and the COM of markers measured in each EPID image collected during a single treatment beam delivery. Image #1 of each EPID series is arbitrarily defined as the baseline distance. The mean, standard deviation and 90th percentile tracking error for each field has been calculated. For comparison with Akimoto et al.^5^ the mean value of the maximum errors has been calculated. The standard deviation (SD) of the mean per patient is an estimate for the SD of the systematic error, Σ. The root mean square (RMS) of the SDs gives the random error, σ.^22^

## 3 RESULTS

### 3.1 Phantom study

**FIGURE 4:**
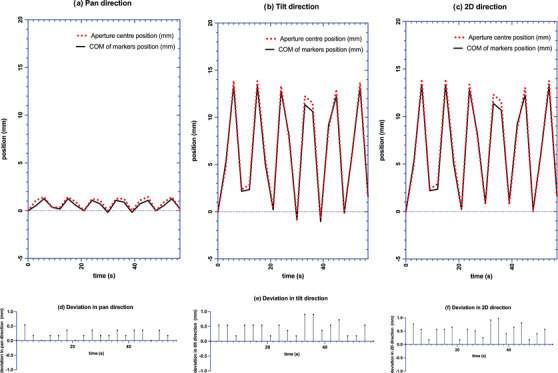
Relative position of aperture center versus COM of markers de‐magnified to isocenter plane in (a) pan (b) tilt and (c) 2D direction versus time for 1 field during the phantom DTT irradiation. (d) E_T_ in pan direction (e) E_T_ in tilt direction (f) E_T_ in 2D direction. Baseline value set to difference in first EPID image.

On the 125 EPID images collected, 100% of the markers were identified. The average ± SD of E_T_ were 0.24 ± 0.21, 0.47 ± 0.38, and 0.58 ± 0.37 mm in pan, tilt and 2D directions, respectively. The 90^th^ percentiles for E_T_ over all fields were 0.54, 1.08, and 1.09 mm in pan, tilt and 2D directions, respectively. Figure 4 shows the relative position of aperture center versus COM of markers in pan and tilt and 2D directions versus time for one field (gantry angle: 0°, ring angle: 355°) during the phantom DTT irradiation.

### 3.2 Patient study

**FIGURE 5:**
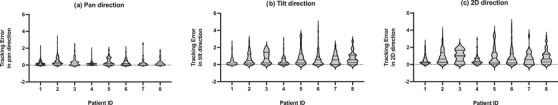
Violin plot of absolute value of tracking error for each patient in (a) pan (b) tilt and (c) 2D direction. Upper horizontal line: 3rd quartile or 75th percentile, Mid horizontal line: median, Lower horizontal line: 1st quartile or the 25th percentile.

**FIGURE 6:**
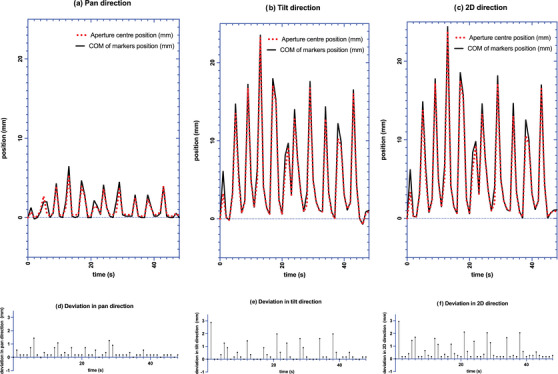
Relative position of aperture center versus COM of markers de‐magnified to isocenter plane in (a) pan (b) tilt and (c) 2D direction versus time for 1 field during the patient 04 ‐ fraction 01 DTT irradiation. (d) E_T_ in pan direction. (e) E_T_ in tilt direction (f) E_T_ in 2D direction. Baseline value set to difference in first EPID image.

Of the 1430 EPID patient images acquired, 78% had detectable markers. Gold markers may not be detected if the images were noisy (due to long beam path or underlying anatomic structure), or if the marker was occluded by the MLC. In total, the E_T_ in eight patients have been calculated. Over all patients, the average ± SD of E_T_ were 0.33 ± 0.41 mm in pan, 0.63 ± 0.75 mm in tilt and 0.77 ± 0.80 mm in 2D directions. In the pan, tilt and 2D direction, over all patients, the 90th percentile for E_T_ were 0.90 mm, 1.75 mm, and 1.90 mm, respectively (Table 2). Figure 5 shows the extended violin plot for the absolute value of E_T_ for each patient in pan, tilt and 2D directions. Figure 6 shows the relative position of aperture center versus COM of markers in pan and tilt and 2D directions versus time for one field (gantry angle: 268°, ring angle: 18°) for patient 04 during DTT irradiation.

**TABLE 2:** Absolute deviations between the measured COM of the markers relative to the aperture‐center position in eight liver patients

	Mean absolute deviation (mm)			SD (mm)			90^th^ percentile (mm)		
Patient ID	Pan	Tilt	2D	Pan	Tilt	2D	Pan	Tilt	2D
1	0.26	0.36	0.52	0.35	0.50	0.54	0.59	0.90	1.27
2	0.46	0.70	0.90	0.49	0.72	0.81	1.08	1.80	2.02
3	0.37	0.84	1.00	0.45	0.68	0.70	0.99	1.80	1.94
4	0.21	0.42	0.53	0.32	0.54	0.59	0.54	0.90	1.14
5	0.42	0.90	1.05	0.45	0.99	1.04	0.94	2.56	2.90
6	0.32	0.75	0.88	0.38	0.97	0.99	0.72	1.80	2.01
7	0.28	0.60	0.70	0.51	0.61	0.76	0.65	1.45	1.52
8	0.31	0.76	0.86	0.41	0.71	0.78	0.97	1.83	1.98
Over all patients	0.33	0.63	0.77	0.41	0.75	0.80	0.90	1.75	1.90
RMS of the SDs random error, σ				0.42	0.73	0.79			
SD of the meansystematic error, Σ	0.08	0.19	0.20						

*Note*: The systematic error, Σ: Standard deviation (SD) of the mean per patient. The random error, σ: Root mean square (RMS) of the SDs.

### 3.3 Planning target margin recipe

Using the methodology from Van Herk,^22^ the random error for all patients was 0.42, 0.73, and 0.79 mm in pan, tilt and 2D directions, respectively. The systematic error for all patients were 0.08, 0.19, and 0.20 mm in pan, tilt and 2D directions, respectively. Using the Van Herk margin formula, the 2D contribution to the planning target margin due to the tracking uncertainty is calculated to be 1.1 mm (assuming 90% patients receive 95% coverage to CTV).

## 4 DISCUSSION

Of the 1430 EPID patient images acquired, 78% had detectable markers. Markers located within 1.24 mm (90th percentile of E_T_) of the field edge may not always appear in the images. In addition, markers may not be visible in the images when they are located under or near high‐density structures (e.g., bone).

Depuydt et al.^20^ evaluated the tracking errors using a 1D moving phantom with sinusoidal motion. They reported that with compensation for system lag, the mean values of the standard deviation over several motion traces were 0.20, 0.22 mm in the pan and tilt directions. The 2D vector modulus overall mean and mean 90^th^ percentile error was 0.25 mm and 0.54 mm, respectively. The phantom study presented here had a mean standard deviation for E_T_ of 0.21 and 0.38 mm in the pan and tilt direction, respectively. The 2D vector modulus overall mean and mean 90^th^ percentile error was 0.58 and 1.09 mm, respectively.

Akimoto et al.^5^ assessed long term stability of tracking errors over a period of 2 years using programmable 1D respiratory motion phantom. They reported that the average value of maximum deviation was 0.38, 0.49, and 0.53 mm in the pan, tilt and 2D direction, respectively. In this paper, the phantom results show the mean value of maximum deviation for E_T_ was 0.50, 1.29, and 1.38 mm in pan, tilt and 2D directions, respectively.

Both Akimoto and Depuydt presented data for one irradiation field. In contrast, this study reports on multiple non‐coplanar fields, representative of a clinical liver SABR delivery, which may result in larger tracking errors.

Poels et al.^18^ reported a gimbaled tracking 90th percentile error of 2.24, 2.81, and 3.45 mm in the pan, tilt and 2D direction, respectively, based on EPID fluoroscopy for one liver cancer patient. Our study of eight liver SABR patients reports a 90th percentile tracking error of 0.90, 1.75, and 1.90 mm in the pan, tilt and 2D directions, respectively. Poels et al. reported the mean ± SD tracking error for their liver patient as 0.32 ± 1.36 and 0.76 ± 1.61 mm, in the pan and tilt direction, respectively. Our study shows for all patients, the average ± SD of E_T_ was 0.33 ± 0.41 mm and 0.63 ± 0.75 mm in the pan and tilt direction.

Other sources of uncertainty introduced into the current EPID measurement study analysis include the accuracy of the field edge and marker localization and physical tracking uncertainty during treatment. The errors presented based on the EPID measurements are likely an overestimate.

There are several sources of uncertainty that contribute to the true error and contribute to the PTV margin specific to DTT:

1) DTT occurs throughout the entire respiratory cycle whereas the planning (in this study) is performed on a single phase (exhale). This is typical for DTT planning optimization as 4D algorithms are not yet available, although attempts to quantify this effect on dosimetry have been published,^23^, ^24^, ^25^ It is possible that the relative position between the target volume and the implanted fiducials may change due to deformations over the respiratory cycle.^26^

2) Migration or deformation of the fiducial relative locations during respiration affecting the center‐of‐mass location during treatment relative to the planned location,^27^

3) Uncertainty in fitting the prediction algorithm which correlates the external breathing trace to the detected fiducial positions during fluoroscopy.^28^

4) Latency between the actual position of the target and the linac DTT beam control. This has been characterized by Depuydt et al.^20^ for the Vero4DRT system.

The 5 mm PTV margin was chosen to be added to the CTV at the time of the initiation of this center's clinical fiducial based DTT program based on the previously published literature discussed above.

## 5 CONCLUSION

DTT beam delivery uncertainty has been evaluated in phantom and in eight liver SABR patients (thirteen fractions) by detecting gold markers visible in collected EPID images. The contribution of this uncertainty to the planning target margin is 1.1 mm. Given other published sources of uncertainty, the current clinical isotropic margin of 5 mm should be sufficient for fiducial based DTT patients at our center and may possibly be reduced once other contributions to the overall uncertainty have been assessed.

## AUTHOR CONTRIBUTION

Study conception and design: AB, MR. EPID Data: all authors. JAVA code preparation: MR, KL. Data Analysis: MR, AB. Manuscript Preparation: MR, AB. Manuscript Revision/Editing: all authors.

## CONFLICTS OF INTEREST STATEMENT

The authors have no relevant conflicts of interest to disclose.
